# Leukadherin-1-Mediated Activation of CD11b Inhibits LPS-Induced Pro-inflammatory Response in Macrophages and Protects Mice Against Endotoxic Shock by Blocking LPS-TLR4 Interaction

**DOI:** 10.3389/fimmu.2019.00215

**Published:** 2019-02-12

**Authors:** Xiaoying Yao, Guanjun Dong, Yuzhen Zhu, Fenglian Yan, Hui Zhang, Qun Ma, Xingqin Fu, Xuehui Li, QingQing Zhang, Junfeng Zhang, Hui Shi, Zhaochen Ning, Jun Dai, Zhihua Li, Chunxia Li, Bo Wang, Jiankuo Ming, Yonghong Yang, Feng Hong, Xiangzhi Meng, Huabao Xiong, Chuanping Si

**Affiliations:** ^1^Institute of Immunology and Molecular Medicine, Jining Medical University, Shandong, China; ^2^School of Medicine and Life Sciences, University of Jinan-Shandong Academy of Medical Sciences, Shandong, China; ^3^Department of Central Laboratory, Affiliated Hospital of Jining Medical University, Jining, Shandong, China; ^4^Department of Microbiology, Immunology and Molecular Genetics, University of Texas Health Science Center at San Antonio, San Antonio, TX, United States; ^5^Department of Medicine, Icahn School of Medicine at Mount Sinai, Immunology Institute, New York, NY, United States

**Keywords:** CD11b, macrophage, endotoxin shock, TLR4, LPS, leukadherin-1 (LA1)

## Abstract

Dysregulation of macrophage has been demonstrated to contribute to aberrant immune responses and inflammatory diseases. CD11b, expressed on macrophages, plays a critical role in regulating pathogen recognition, phagocytosis, and cell survival. In the present study, we explored the effect of leukadherin-1 (LA1), an agonist of CD11b, on regulating LPS-induced pro-inflammatory response in macrophages and endotoxic shock. Intriguingly, we found that LA1 could significantly reduce mortalities of mice and alleviated pathological injury of liver and lung in endotoxic shock. *In vivo* studies showed that LA1-induced activation of CD11b significantly inhibited the LPS-induced pro-inflammatory response in macrophages of mice. Moreover, LA1-induced activation of CD11b significantly inhibited LPS/IFN-γ-induced pro-inflammatory response in macrophages by inhibiting MAPKs and NF-κB signaling pathways *in vitro*. Furthermore, the mice injected with LA1-treated BMDMs showed fewer pathological lesions than those injected with vehicle-treated BMDMs in endotoxic shock. In addition, we found that activation of TLR4 by LPS could endocytose CD11b and activation of CD11b by LA1 could endocytose TLR4 *in vitro* and *in vivo*, subsequently blocking the binding of LPS with TLR4. Based on these findings, we concluded that LA1-induced activation of CD11b negatively regulates LPS-induced pro-inflammatory response in macrophages and subsequently protects mice from endotoxin shock by partially blocking LPS-TLR4 interaction. Our study provides a new insight into the role of CD11b in the pathogenesis of inflammatory diseases.

## Introduction

Endotoxic shock, characterized by poor tissue perfusion and multi-organ dysfunction, is a fatal condition caused by a disordered immune response to bacterial infection (1). Macrophages, a highly heterogeneous population of cells derived from the myeloid cell lineage, are key cells involved in the pathogenesis of endotoxic shock (2–4). Macrophages are activated in response to various stimuli and acquire distinct functional abilities; they are classified into two categories: pro-inflammatory M1 (classically activated macrophages) and anti-inflammatory M2 (alternatively activated macrophages) (5–7). M1 macrophages produce high levels of pro-inflammatory cytokines, such as TNF-α, IL-1β, IL-6, and IL-12 (8), whereas M2 macrophages exhibit mannose receptors (MRC1/CD206), and produce high levels of anti-inflammatory cytokines, such as IL-10 and arginase-1 (Arg-1) (9). In the early stages of endotoxin shock, bacterial LPS binds to LPS binding protein (LBP) and forms the LPS/LBP complex, which transfers LPS to CD14 (10, 11). Subsequently, CD14 transfers LPS to the TLR4/MD-2 complex and mediates TLR4 internalization, which dimerizes and triggers MyD88- and TRIF-dependent production of pro-inflammatory cytokines, and type I interferons (12–17). These studies proved that macrophages are activated in the early stages of endotoxin shock, and then lead to tissue damage by inducing increased production of pro-inflammatory cytokines. Thus, there is an urgent need to investigate the underlying regulatory mechanisms of LPS-induced pro-inflammatory response in macrophages during endotoxic shock.

In fact, several molecules have been shown to act as co-receptors and/or accessory molecules to regulate LPS-activated signaling pathway. One of the most important regulators is CD11b/CD18. CD11b/CD18, which is a heterodimer of the αM (CD11b) and β2 (CD18) subunits, and is extensively expressed in most immune cells, including macrophages, dendritic cells (DCs), and neutrophils (18–21). CD11b regulates a broad range of immune responses by modulating pathogen recognition, phagocytosis, and cell survival (20, 22, 23). Deficiency in CD11b exacerbates dextran sodium sulfate–induced colitis and blocks oral tolerance by polarizing the differentiation of interleukin 17-producing helper T cells (24, 25), which indicates their involvement in tolerance maintenance. However, the role of CD11b in regulation of TLR-triggered innate immune responses remains controversial. On one hand, some studies have reported that CD11b acts as a negative regulator of TLR signaling pathway (26–28), while some studies have shown that CD11b promoted optimal TLR-mediated response (22, 29). Thus, the role of CD11b in the regulation of LPS-induced activation of M1 macrophages needs to be further elucidated.

In the present study, we found that LA1, a small molecule agonist of CD11b, reduced mortalities of mice and alleviated pathological injury of liver and lung in LPS-induced endotoxic shock. Moreover, LA1-mediated activation of CD11b significantly inhibited LPS-induced pro-inflammatory response in macrophages *in vivo* and *in vitro*. Mice injected with LA1-treated BMDMs showed reduced pathological lesions than mice injected with vehicle-treated BMDMs when treated with LPS. In addition, activation of TLR4 by LPS could endocytose CD11b and activation of CD11b by LA1 could endocytose TLR4 *in vitro* and *in vivo*, subsequently blocking the binding of LPS and TLR4 partially. Taken together, our study reveals a crucial role of CD11b, in the regulation of LPS-induced pro-inflammatory response in macrophages, and subsequently, protection of mice from endotoxic shock, thereby providing novel insights into the role of CD11b in the pathogenesis of inflammatory diseases.

## Materials and Methods

### Mice

C57BL/6 mice were purchased from Peng Yue experimental animal breeding company (Jinan, China) and maintained under specific pathogen-free conditions at Jining Medical University. Female mice between the ages of 6–8 weeks were used for study. All animal procedures were conducted in accordance with institutional guidelines for animal care and approved by Animal Care Committee at Jining Medical University.

### Preparation of Bone Marrow-Derived Macrophages

Bone marrow (BM) cells, isolated from tibias and femurs of C57BL/6 mice were cultured in complete DMEM supplemented with GM-CSF (10 ng/ml; PeproTech, USA). On day 4, half the amount of medium was replaced by fresh DMEM supplemented with GM-CSF (10 ng/ml). On day 7, bone marrow-derived macrophages (BMDMs) were harvested, and then, seeded in fresh complete DMEM at a density of 2 × 10^6^ cells/ml for experiments. BMDMs were administered with LPS (200 ng/ml; Sigma-Aldrich, USA) and IFN-γ (10 ng/ml; PeproTech, USA) or administered with LA1 (20 μM; Selleck, USA) for different times to detect the expression of TLR4 and CD11b. Under certain conditions, BMDMs were pretreated with LA1 (20 μM) for 2 h. Cells were stimulated with LPS (200 ng/ml) and IFN-γ (10 ng/ml) to detect pro-inflammatory response in macrophages: the mRNA levels of pro-inflammatory cytokines were detected at 6 h; the secretion of pro-inflammatory cytokines and CD86 expression were detected at 24 h; the phosphorylation levels of p38, ERK1/2, JNK, and p65 were detected at 30 and 60 min.

### Preparation of Bone Marrow-Derived Dendritic Cells

BM cells were cultured in complete RPMI 1640 medium supplemented with GM-CSF (20 ng/ml) and IL-4 (10 ng/ml). Every other day, half the amount of medium were replaced by RPMI 1640 medium and supplemented with GM-CSF (20 ng/ml) and IL-4 (10 ng/ml). On day 7, bone marrow-derived dendritic cells (BMDCs) were harvested and then seeded in fresh complete RPMI 1640 medium at a density of 2 × 106 cells/ml for experiments.

### TLR4 and CD11b Measurement in Mice

C57BL/6 mice (female, 6- to 8-week-old) were randomly divided into different groups. The mice were administered either LPS (10 μg/g of body weight) or LA1 (40 μg/g of body weight) for different times. The expressions of TLR4 and CD11b were detected by flow cytometry in peripheral blood mononuclear cells (PBMCs), peritoneal cells or splenocytes.

### Murine Model of Endotoxic Shock

C57BL/6 mice (female, 6- to 8-week-old) were randomly divided into different groups. C57BL/6 mice were pretreated for 2 h with either LA1 at 10, 20, and 40 μg/g of body weight or vehicle, administered by intraperitoneal injection. For flow cytometry and histological analyses, mice were intraperitoneally injected with LPS at 10 μg/g of body weight, followed by collection of peritoneal cells and spleens after 12 h. Livers and lungs were collected as well for hematoxylin and eosin staining. Survivals after administration of LPS (25, 37.5, or 50 μg/g of body weight) were monitored. For collection of serum and peritoneal cells, mice were injected with LPS (10 μg/g of body weight), followed by collection of serum and peritoneal cells after 6 h.

### Adoptive Transfer and LPS-Induced Endotoxin Shock

C57BL/6 mice received i.p. injections of either vehicle-treated BMDMs or LA1-treated BMDMs (2 × 10^6^ cells/mouse). After 12 h, mice were injected with LPS (10 μg/g of body weight), and serums were collected after 6 h. Livers and lungs were collected after 12 h as well for hematoxylin and eosin staining.

### RNA Isolation and Quantitative Real-Time RT–PCR (qRT-PCR)

Total RNA was extracted using TRIzol reagent (Invitrogen) according to the manufacturer's instructions. qRT-PCR assays for mRNA were carried out using SYBR Green PCR Master Mix (Vazyme Biotech, China). The following sequence-specific primers were synthesized: 5′-GAAATGCCACCTTTTGACAGTG-3′ and 5′-TGGATGCTCTCATCAGGACAG-3′ for mouse IL-β, 5′-CCAGAAACCGCTATGAAGTTCCT-3′ and 5′-CACCAGCATCAGTCCCAAGA-3′ for mouse IL-6, 5′AGACATGGAGTCATAGGCTCTG-3′ and 5′-CCATTTTCCTTCTTGTGGAGC A-3′ for mouse IL-12, 5′-GCCACCACGCTCTTCTGTCT-3′ and 5′-GGTCTGGG CCATAGAACTGATG-3′ for TNF-α, and 5′-AACGACCCCTTCATTGAC-3′ and 5′-TCC ACGACATACTCAGCAC-3′ for mouse GAPDH. The 2-ΔΔCt method was used for real-time quantitative PCR gene expression analysis. All data were normalized with the GAPDH level.

### Enzyme-Linked Immunosorbent Assay

The concentrations of IL-6, IL-12, IL-1β, and TNF-α in mice serum and in culture supernatant of BMDMs or BMDCs were determined using the mouse ELISA kit (Biolegend, USA). Briefly, capture antibody was coated directly onto 96-well ELISA plates and incubated overnight at 4°C. Then, the plates were washed four times by phosphate buffered saline (PBS, containing Tween 20) and were blocked with 200 μl Assay Diluent (1% BSA) to avoiding non-specific binding. Then, 100 μl of each sample were added to the well. After incubation at 25°C for 2 h, the protein was removed, and the wells were washed with PBS Tween 20 for 4 times. Then, we added 100 μl biotinylated antibodies to each well and incubated at 25°C for 1 h. After washing for 4 times, we added 100 μl streptavidin-HRP in each well and incubated at 25°C for 30 min. TMB (100 μl) was added to each well after washing for five times. After appropriate time, we added 100 μl stop solution to each well and the plates were read at 450 nm using a microplate reader (BioTek). Linearity was in the range of 0–500 or 0–2,000 pg/ml. All samples were assayed in duplicates.

### Flow Cytometry

For phenotype staining, cells were washed twice with PBS. The cells were stained with mouse antibodies of CD11b (FITC-labeled, eBioscience, USA), F4/80 (PE-labeled, eBioscience, USA), F4/80 (FITC-labeled, eBioscience, USA), CD86 (APC-labeled, eBioscience, USA), TLR4 (APC-labeled, eBioscience, USA), CD11c (FITC-labeled, eBioscience, USA), CD11c (PE-labeled, eBioscience, USA), and CD40 (PE-labeled, eBioscience, USA) for 30 min at 4°C according to the manufacturer's instructions. Cells were analyzed by FACS Calibur (Becton Dickinson, USA). An isotype control was used for each antibody.

### Immunoblotting Analysis

Cell lysates were quantified by BCA method, and then, resolved using 10% SDS-PAGE followed by transfer onto PVDF membranes (Millipore) for 2 h at 100 V with a standard transfer solution. After being blocked with 3% BSA, membranes were incubated with primary antibodies for p38 (1:1,000, CST, USA), p-p38 (1:1,000, CST, USA), p65 (1:1,000, CST, USA), p-p65 (1:1,000, CST, USA), JNK (1:1,000, CST, USA), p-JNK (1:1,000, CST, USA), ERK1/2 (1:1,000, CST, USA), and p-ERK1/2 (1:1,000, CST, USA), and with β-actin antibody (1:1,000, CST, USA) at 4 °C overnight followed by incubation with secondary antibody HRP-labeled Goat Anti-Rabbit (1:3,000, Beyotime Biotechnology, China) or HRP-labeled Rabbit Anti-Mouse (1:3,000, Beyotime Biotechnology, China). ECL kit (Thermo, USA) with chemiluminescence was used for detection. β-actin was used as an internal control.

### Histological Analyses

Sections (4 μm) were cut from paraffin-embedded lung and liver tissues, fixed in paraformaldehyde (Sigma, USA), and stained with hematoxylin and eosin. The degree of lung injury was scored based on the following variables: hemorrhage, lung edema, inflammatory cell infiltration, hyaline membrane, and atelectasis. The degree of each abnormality was graded numerically from 0 (normal) to 4 (diffuse injury) according to the following criteria (19): no injury = 0, injury to 25% of the field = 1, injury to 50% of the field = 2, injury to 75% of the field = 3, and diffuse injury = 4. The liver pathological injury was expressed as the sum of the individual score grades, 0 (no findings), 1 (mild), 2 (moderate), and 3 (severe), for each of the following six parameters: cytoplasmic color fading, vacuolization, nuclear condensation, nuclear fragmentation, nuclear fading, and erythrocyte stasis. Blind analysis was performed to determine the lesion degree of all samples.

### *In Situ* Terminal Deoxynucleotidyl Transferase –Mediated Uridine Triphosphate Nick-End Labeling Assay

Apoptosis of pneumonocyte and hepatocytes were detected by transferase-mediated uridine triphosphate nick-end labeling (TUNEL) assay, which was performed according to the manufacturer's protocol (Roche, Switzerland).

### Immunofluorescence Staining and Confocal Microscopy

BMDMs were cultured on coverslips for 7 days before staining. After stimulation with either LPS/IFN-γ or LA1 for different time points, the cells were washed with PBS for three times. Then, slides harboring BMDMs were fixed with immunol staining fix solution (15 min; 25°C) and permeabilized with immunostaining permeabilization buffer with Triton X-100 (20 min; 25°C). After blocking with 1% bovine serum albumin (BSA), BMDM sliders were exposed to primary antibodies overnight at 4°C. Next day, the slides were washed in PBS containing 0.1% Tween 20 for five times, and then, exposed to fluorochrome-labeled secondary antibodies for 1 h (25°C). After PBST washing, DAPI was added to the cells for staining the nucleus. Finally, the cover slips were sealed with an anti-fluorescence quenching agent.

### Co-immunoprecipitation

The BMDMs were challenged with LA1 for 1 or 2 h. Then, the cells were lysed in a modified RIPA buffer containing 1 mM PMSF and 1 × protease inhibitor cocktail, and centrifuged at 12,000 rpm (4°C) for 10 min. One aliquot of the supernatant was saved as the input control, the remainder was separately incubated with anti-CD11b antibody (abcam, USA), anti-TLR4 antibody (cell signaling technology, USA) and negative control IgG antibody (Beyotime, China) followed by pull-down with 30 μl Protein A Agarose beads (cell signaling technology, USA). The beads were then collected by centrifugation at 12,000 rpm for 2 min and washed three times with cold PBS. The immunoprecipitates were eluted by boiling in 1 × loading buffer for 10 min and subjected to western blot analysis along with input sample as described above.

### Statistical Analysis

All values in the graphs were given as means plus or minus standard error of the mean (SEM). Data were using Student's *T*-test, one-way analysis of variance (ANOVA) *post-hoc* Tukey Multiple Comparison Test or two-way ANOVA *post-hoc* Bonferroni Multiple Comparison Test. Kaplan–Meier method was used to estimate overall survivals and the Log-rank test was applied to determine the differences of survival rate. *P* < 0.05 were considered significant.

## Results

### Activation of CD11b by LA1 Reduced LPS-Induced Mortality

**Figure 1:**
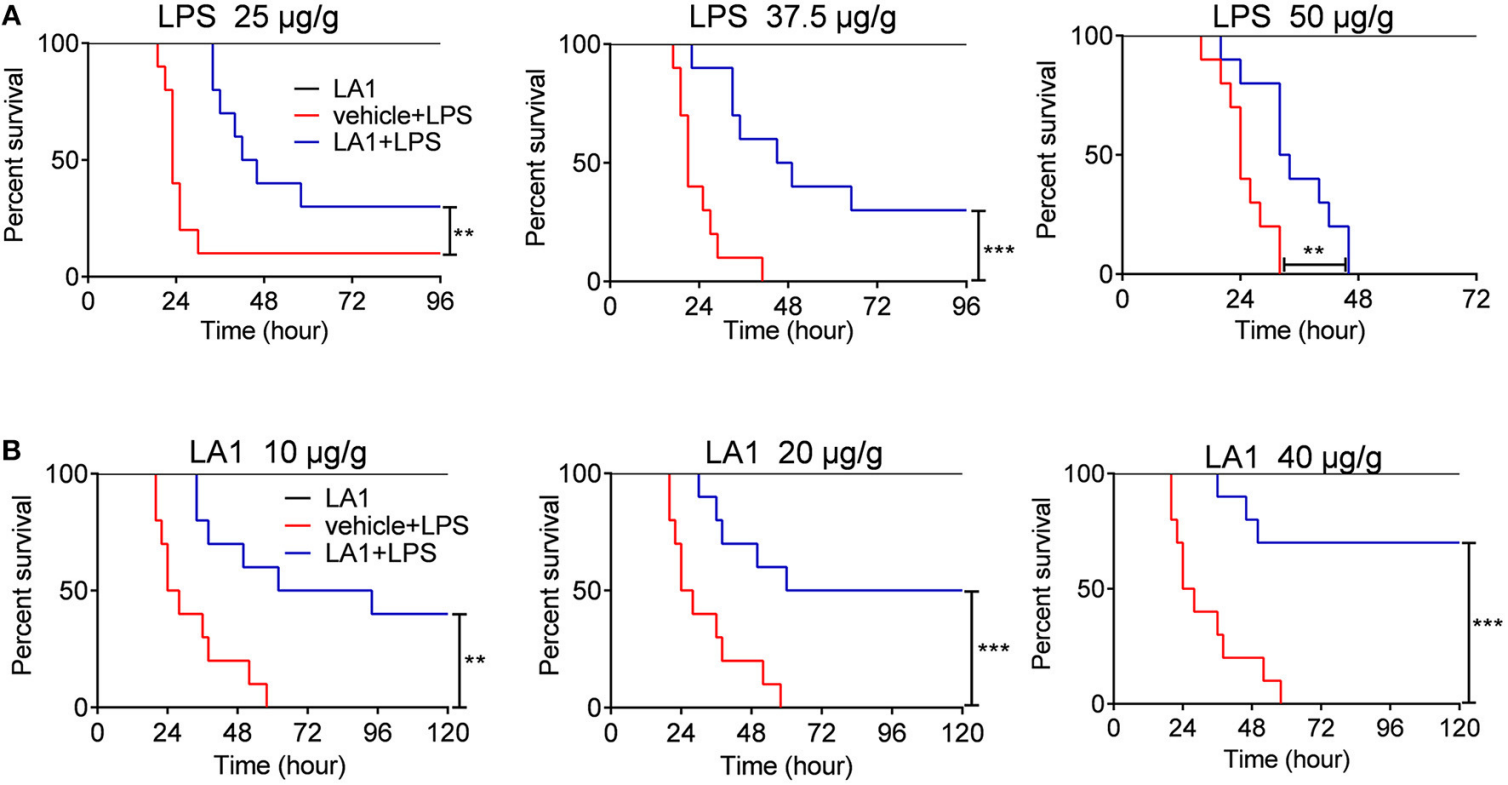
Activation of CD11b by LA1 reduced LPS-induced mortality. **(A)** C57BL/6 mice were treated with either LA1 (40 μg/g of body weight) or vehicle followed by LPS stimulation (25, 37.5, and 50 μg/g of body weight). The survivals of mice were observed (*n* = 10 mice/group) **P < 0.01, ***P < 0.001. Kaplan–Meier method was used to estimate overall survivals and the survival rates were determined by Log-rank test. **(B)** C57BL/6 mice were treated with either LA1 (10, 20, and 40 μg/g of body weight) or vehicle followed by LPS (37.5 μg/g of body weight) stimulation. The survivals of mice were observed (*n* = 10 mice/group) **P < 0.01, ***P < 0.001. Kaplan–Meier method was used to estimate overall survivals and the survival rates were determined by Log-rank test.

Up to date, the role of CD11b in regulation of innate immune responses remains controversial. Some data indicate that CD11b inhibits the development of inflammatory diseases (19, 28), while others showed that CD11b-deficient mice were more resistant to inflammatory diseases (22). We found that silencing of CD11b inhibited the pathogenesis of LPS-induced endotoxin shock and the pro-inflammatory response of macrophages and DCs (Figure S1), suggesting that CD11b participates in the pathogenesis of endotoxic shock. In this study, we focused on LA1, an agonist of CD11b, and attempted to comprehend the effects of LA1 on LPS-induced pro-inflammatory response in macrophages and the pathogenesis of endotoxic shock. Mice were administered with either vehicle or LA1 at a dose of 40 μg/g followed by stimulation with different doses of LPS and the mortalities of mice were observed. As shown in Figure 1A, LA1 reduced the mortalities of mice induced by different doses of LPS. Moreover, mice were administered with either vehicle or different doses of LA1 followed by LPS stimulation, and the mortalities of mice were observed. Mice treated with different doses of LA1 showed significantly reduced LPS-induced mortalities (Figure 1B). The data revealed that LA1 mitigated LPS-induced mortality in mice.

### Activation of CD11b by LA1 Alleviated LPS-Induced Lung and Liver Injury

**Figure 2:**
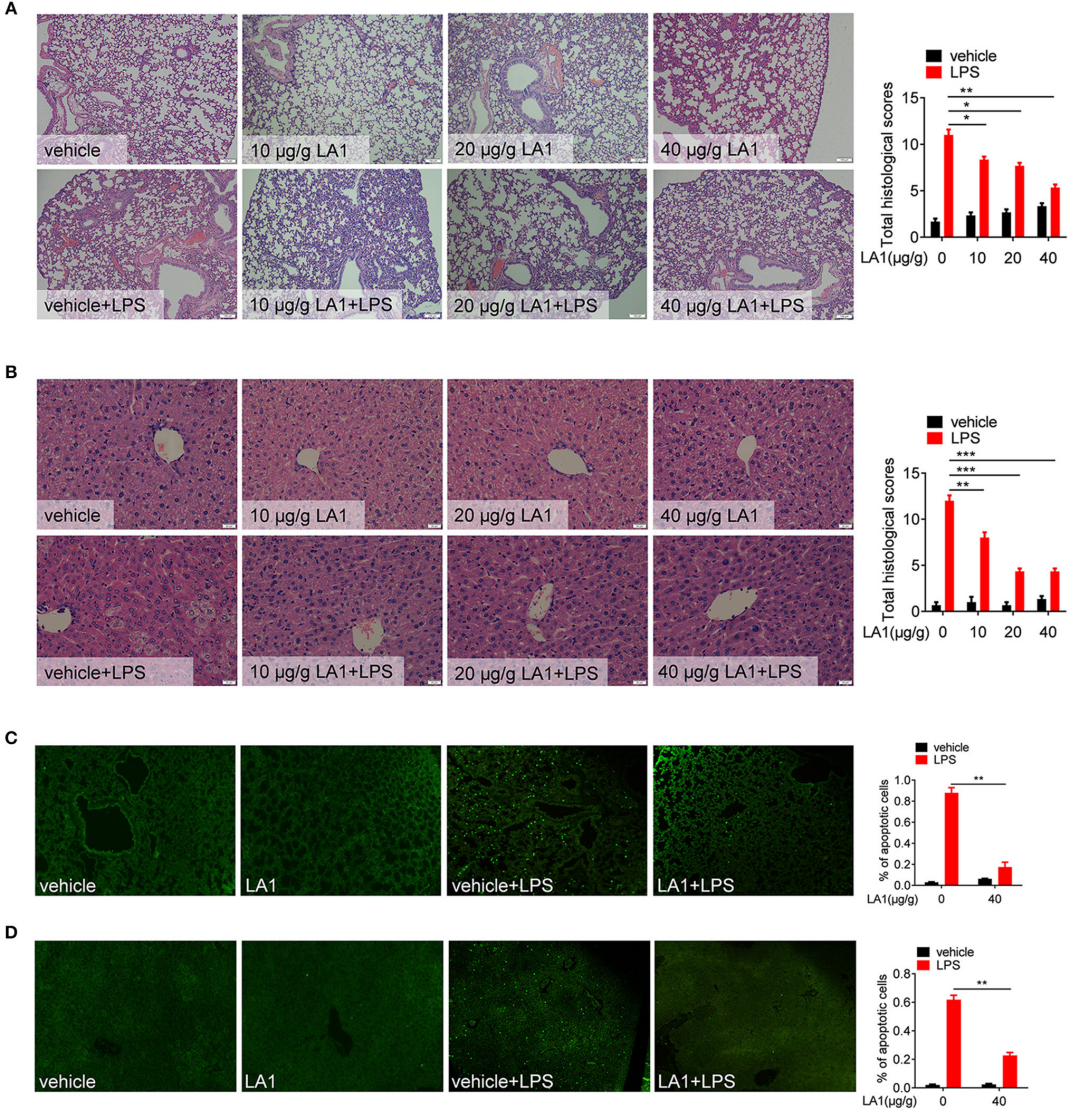
Activation of CD11b by LA1 alleviated LPS-induced lung and liver injury. **(A, B)** C57BL/6 mice were treated with either LA1 (10, 20, and 40 μg/g of body weight) or vehicle for 2 h, and then, stimulated with LPS (10 μg/g of body weight). After 12 h, tissues of lung and liver were fixed with 4% paraformaldehyde and paraffin-embedded lung **(A)** and liver **(B)** sections were stained with H&E. **(C, D)** C57BL/6 mice were treated with either LA1 (40 μg/g of body weight) or vehicle for 2 h, and then, stimulated with LPS (10 μg/g of body weight). After 12 h, apoptosis in the cells of lung **(C)** and liver **(D)** was analyzed by TUNEL. Data shown are representative of three independent experiments. Error bars represent S.E.M. *p < 0.05, **p < 0.01, ***p < 0.001, as determined by two-way ANOVA *post-hoc* Bonferroni Multiple Comparison Test.

Next, the effects of LA1 on the pathological alterations were observed. Mice were pretreated with different doses of LA1 for 2 h followed by LPS administration for 12 h, and then, the pathological lesions were observed. As expected, LA1 treatment significantly alleviated the damage to lung and liver in mice stimulated with LPS (Figures 2A,B). Moreover, TUNEL staining of the lung tissue and liver tissue revealed that LA1-treated mice displayed a lower percentage of apoptotic cells compared to control group (Figures 2C,D). Based on these data, we demonstrated that activation of CD11b by LA1 protected mice from pathological alterations, which induced by endotoxic shock, indicating that CD11b might act as a potential therapeutic target for the treatment of inflammatory diseases.

### Activation of CD11b by LA1 Inhibited LPS-Induced Pro-Inflammatory Response in Macrophages *in vivo*

**Figure 3:**
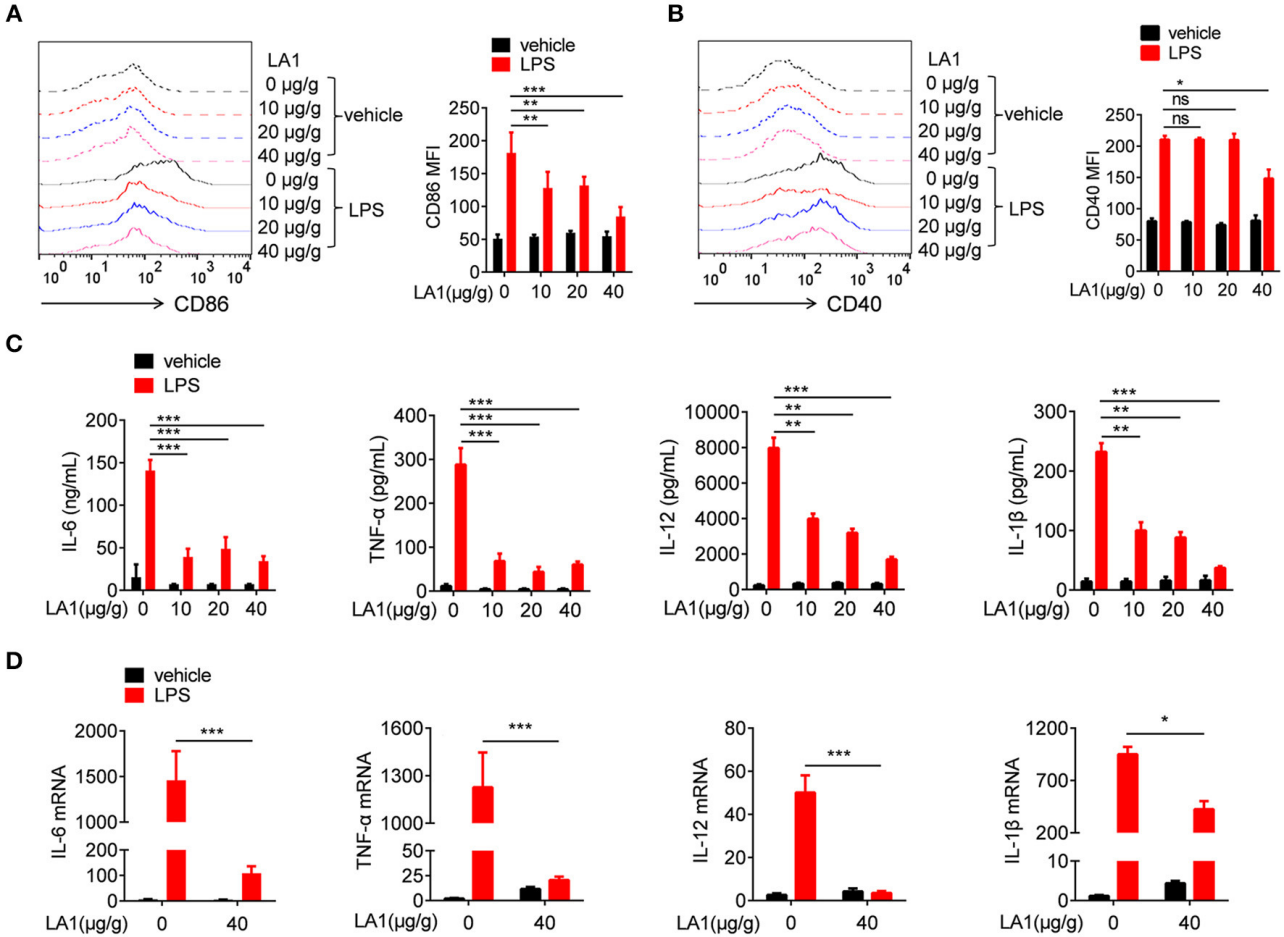
Activation of CD11b by LA1 inhibited LPS-induced pro-inflammatory response in macrophages *in vivo*. C57BL/6 mice were treated with either LA1 (10, 20, and 40 μg/g of body weight) or vehicle for 2 h, and then, stimulated with LPS (10 μg/g of body weight). FACS analysis was performed to assess the expressions of CD86 **(A)** and CD40 **(B)** in splenic macrophages after 12 h. Levels of IL-6, TNF-α, IL-12, and IL-1β in serum were determined by ELISA after 6 h **(C)**. The expressions of IL-6, TNF-α, IL-12, and IL-1β **(D)** in peritoneal cells were examined by qRT-PCR after 6 h. Data are shown as the means ± SEM (*n* = 6 replicates) and are representative of three independent experiments. Error bars represent S.E.M. *p < 0.05, **p < 0.01, ***p < 0.001, as determined by two-way ANOVA *post-hoc* Bonferroni Multiple Comparison Test; ns denotes *p* > 0.05.

It is common knowledge that macrophages play a momentous role in the early stage of endotoxic shock by secreting high amounts of pro-inflammatory cytokines (30). To investigate whether the effect exhibited by LA1 on endotoxic shock was due to inhibition of LPS-induced pro-inflammatory response in macrophages, we first examined the expressions of CD86 and CD40 in macrophages obtained from either LA1 or vehicle-treated mice after LPS treatment. As shown in Figures 3A,B, compared with vehicle-treated mice, mice administered with LA1 exhibited significantly reduced expressions of CD86 and CD40 in macrophages. The expressions of CD86 and CD40 in dendritic cells were also detected. Consistently, LA1 treatment significantly reduced the expressions of CD86 and CD40 in dendritic cells (Figure S2). In addition, we also detected the levels of pro-inflammatory cytokines in the serum and peritoneal cells of mice. Compared with vehicle-treated mice, mice administered with LA1 showed significantly reduced serum levels of IL-6, TNF-α, IL-12, and IL-1β (Figure 3C). qRT-PCR analysis also showed that LA1 treatment significantly reduced the mRNA levels of IL-6, TNF-α, IL-12, and IL-1β in cells from peritoneal cavity of mice stimulated with LPS (Figure 3D). All these data demonstrated that activation of CD11b by LA1 significantly inhibited LPS-induced pro-inflammatory response in macrophages *in vivo*.

### Activation of CD11b by LA1 Inhibited LPS/IFN-γ-Induced Pro-Inflammatory Response in Macrophages *in vitro*

**Figure 4:**
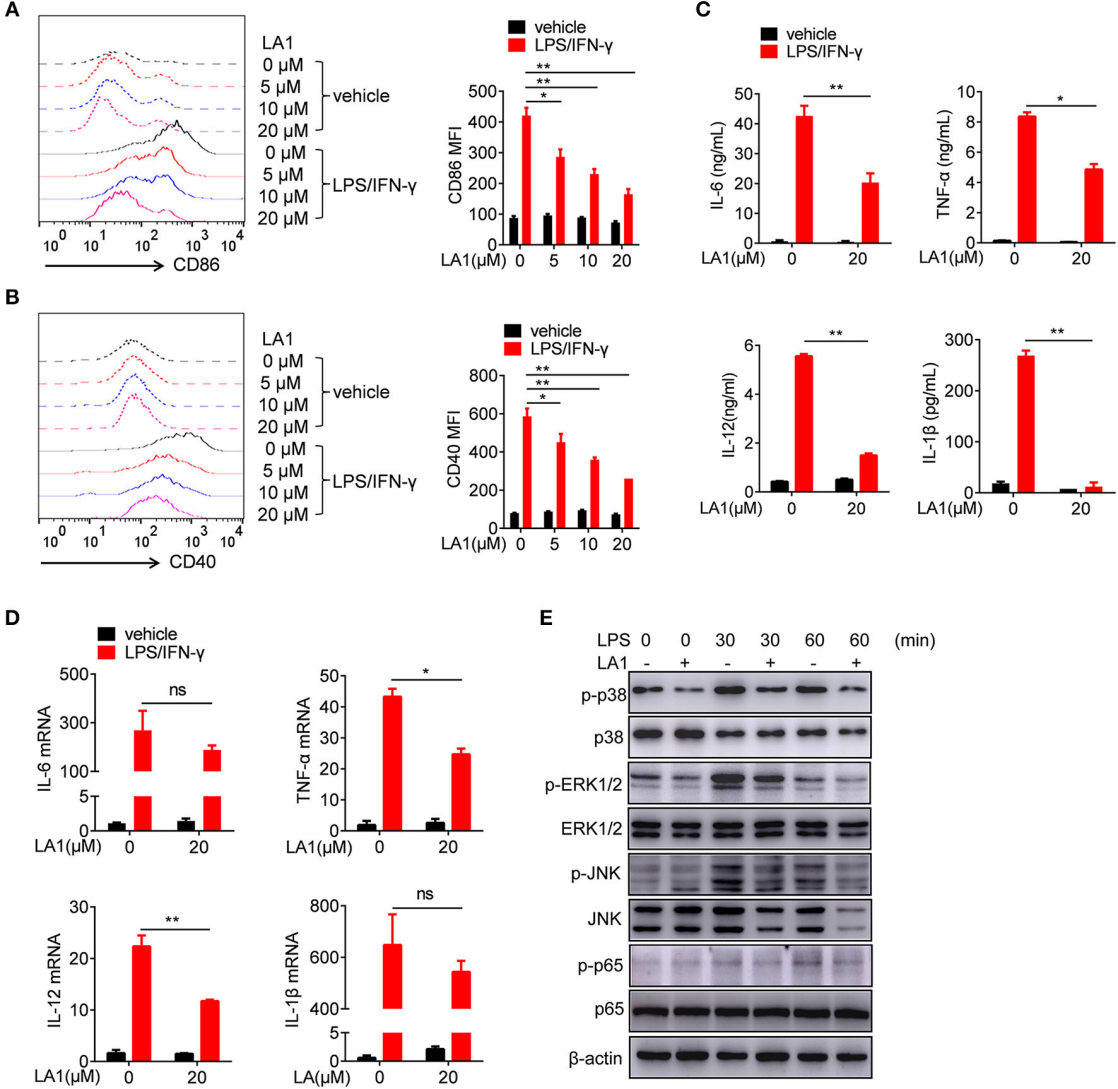
Activation of CD11b by LA1 inhibited LPS/IFN-γ-induced pro-inflammatory response in macrophages *in vitro*. **(A–D)** BMDMs were pretreated with LA1 (0, 5, 10, and 20 μM) for 2 h, and then, the cells were treated with LPS (200 ng/ml) and IFN-γ (10 ng/ml). FACS analysis was performed to assess the expressions of CD86 **(A)** and CD40 **(B)** after 24 h. Levels of IL-6, TNF-α, IL-12, and IL-1β in supernatant were determined by ELISA after 24 h **(C)**. Expressions of IL-6, TNF-α, IL-12, and IL-1β were measured using qRT-PCR after 6 h **(D)**. **(E)** BMDMs were pretreated with either 20 μM LA1 or vehicle for 2 h, and then, the cells were stimulated with LPS (200 ng/ml) and IFN-γ (10 ng/ml) for 30 and 60 min. Cell lysates were prepared and subjected to immunoblotting with the indicated antibodies. β-actin was used as loading control. Data are representative from one out of three biological replicates, each with three technical replicates. Error bars represent S.E.M. *p < 0.05, **p < 0.01, as determined by two-way ANOVA *post-hoc* Bonferroni Multiple Comparison Test; ns denotes *p* > 0.05.

To further investigate the role of CD11b in regulation of LPS-induced pro-inflammatory response in macrophages, we used LA1 to activate CD11b, and examined its effect on LPS/IFN-γ-induced pro-inflammatory response in macrophages. CCK8 analysis and microscopic observation of cell morphology revealed that LA1, below the concentration of 20 μM, had no effect on cell viability (Figure S3). Therefore, the concentrations of LA1 used for subsequent experiments were all below 20 μM. To further investigate the effect of LA1 on LPS-induced pro-inflammatory response, BMDMs were pretreated with different doses of LA1 for 2 h followed by LPS/IFN-γ stimulation. LA1 treatment significantly inhibited LPS/IFN-γ-induced expressions of CD86 (Figure 4A) and CD40 (Figure 4B). Moreover, compared with non-treated BMDMs, BMDMs treated with LA1 showed significantly reduced levels of IL-6, TNF-α, IL-12, and IL-1β after LPS/IFN-γ stimulation (Figure 4C). To further investigate this phenomenon, mRNA levels of these inflammatory factors were detected in BMDMs treated with 20 μM LA1. The changes in mRNA expressions were in consistence with the changes in protein expressions (Figure 4D). In addition, the effect of CD11b on LPS/IFN-γ-induced activation of dendritic cells was also investigated. Consistent with our speculations, LA1 inhibited the protein as well as the mRNA levels of LPS/IFN-γ-induced secretion of IL-6, TNF-α, IL-12, and IL-1β in BMDCs (Figure S4). Therefore, these data proved that activation of CD11b by LA1 negatively regulated LPS-induced pro-inflammatory response in macrophages.

As is already known, LPS activates TLR4, and subsequently, activates MAPKs and NF-κB signaling pathways (27, 28). Thus, we examined the effects of LA1 on the activation of NF-kB and MAPKs pathways induced by LPS/IFN-γ. BMDMs were pretreated with either LA1 or vehicles followed by LPS/IFN-γ stimulation, and then, the activation of MAPKs and NF-κB signaling pathways were detected. Western blot analyses confirmed that LA1 suppressed LPS/IFN-γ-induced phosphorylation of p38, ERK1/2, JNK, and p65 (Figure 4E), indicating that CD11b negatively regulated LPS-induced activation of NF-κB and MAPKs pathways. Together, these data demonstrated that CD11b negatively regulated LPS-induced pro-inflammatory response in macrophages by inhibiting the activation of MAPKs and NF-κB signaling pathways.

### LPS-Induced Inflammation Was Reduced in Mice Transferred With LA1- Treated BMDMs Compared With Vehicle-Treated BMDMs

**Figure 5:**
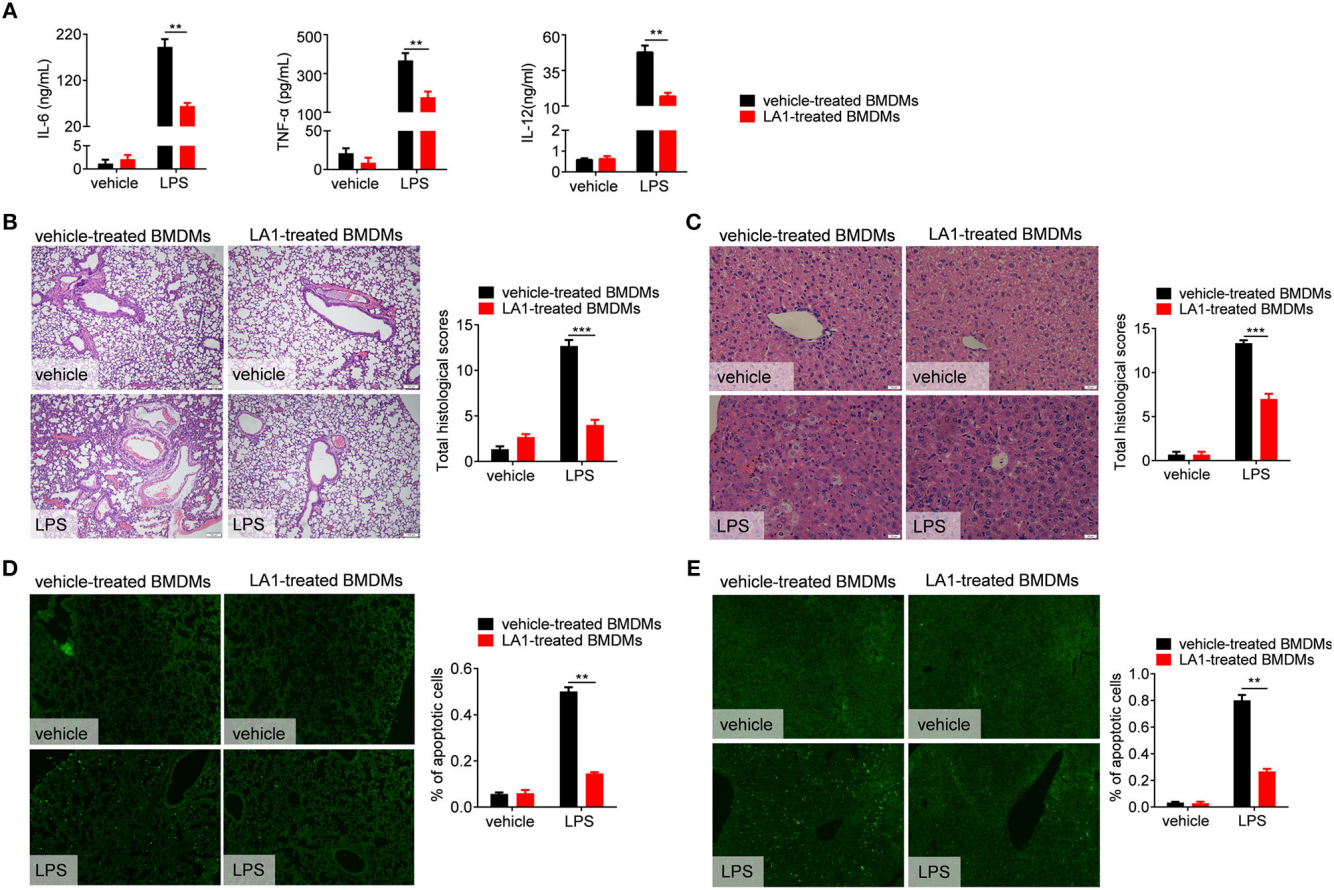
LPS-induced inflammation was reduced in mice transferred with LA1- treated BMDMs compared with vehicle-treated BMDMs. BMDMs were treated with either LA1 (20 μM) or vehicle for 2 h before being injected into C57BL/6 mice (i.p.). After 12 h, the recipient mice were stimulated with LPS (10 μg/g of body weight). **(A)** Serum levels of IL-6, TNF-α, IL-12, and IL-1β were determined by ELISA after 6 h. Paraffin-embedded lung **(B)** and liver **(C)** sections were stained with H&E after 12 h. Apoptosis in the cells of lung **(D)** and liver **(E)** were analyzed by TUNEL. Error bars represent S.E.M. **p < 0.01, ***p < 0.001, as determined by two-way ANOVA *post-hoc* Bonferroni Multiple Comparison Test.

Our results have shown that LA1 inhibited LPS-induced pro-inflammatory response in macrophages *in vitro* and *in vivo*. To further validate these results, *in vivo* studies were constructed. BMDMs, pretreated with LA1 or vehicle for 2 h, were intraperitoneally injected into mice followed by LPS administration. As shown in Figure 5A, mice administered with LA1-treated BMDMs showed a significant reduction in the serum levels of IL-6, TNF-α, and IL-12, which play significant roles in cytokine storm–mediated septic shock, than those administered with vehicle-treated BMDMs. Consistently, H&E staining also showed that the damage to lung and liver were remissive in mice administered with LA1-treated BMDMs compared with mice administered with vehicle-treated BMDMs (Figures 5B,C). To further validate these results, TUNEL staining was performed on mouse liver and lung tissues. As shown in Figures 5D,E, mice administered with LA1-treated BMDMs showed a significant reduction in percentage of apoptotic cells in lung and liver compared with mice administered with vehicle-treated BMDMs. All these results demonstrated that activation of CD11b by LA1 inhibited LPS-induced pro-inflammatory response in macrophages and, in turn, protected mice from endotoxic shock.

### Activation of TLR4 by LPS Could Endocytose CD11b in Macrophages

**Figure 6:**
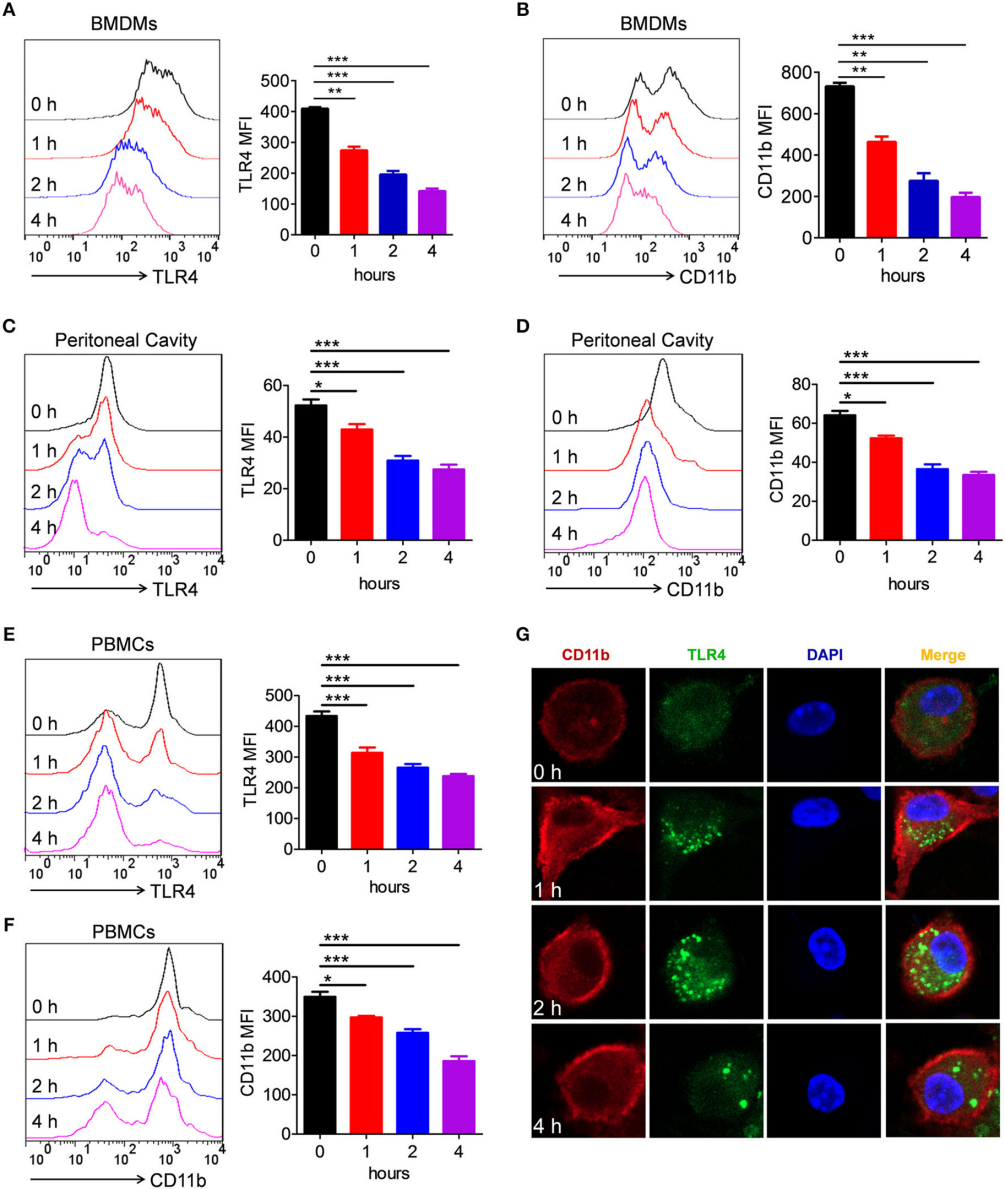
Activation of TLR4 by LPS could endocytose CD11b in macrophages. **(A, B)** BMDMs were treated with LPS (200 ng/ml) and IFN-γ (10 ng/ml) for 1, 2, and 4 h. FACS analysis was performed to assess the expressions of TLR4 **(A)** and CD11b **(B)** in BMDMs. **(C–F)** C57BL/6 mice were stimulated by LPS (10 μg/g of body weight) for 1, 2, and 4 h. FACS analysis was performed to assess the expressions of TLR4 **(C)** and CD11b **(D)** in peritoneal cavity, as well as to assess the expressions of TLR4 **(E)** and CD11b **(F)** in PBMCs. **(G)** BMDMs containing slides were treated with LPS (200 ng/ml) and IFN-γ (10 ng/ml) for 1, 2, and 4 h, and then, the cells were fixed and permeabilized. After staining with anti-TLR4 mAb and anti-CD11b mAb, cells were incubated with an Alexa Fluor 488- and 647-conjugated secondary antibody and analyzed by fluorescent staining. Red represents CD11b; green represents TLR4; light blue represents nuclei with DAPI; and yellow represents merged TLR4 and CD11b. For *in vitro* studies, the data represent one out of the three biological replicates, each with three technical replicates. The data are shown as the means ± SEM (*n* = 6 replicates) and are representative of three independent experiments. Error bars represent S.E.M. *p < 0.05, **p < 0.01, ***p < 0.001, as determined by one-way analysis of variance (ANOVA) *post-hoc* Tukey Multiple Comparison Test.

It is well-known that LPS induced TLR4 endocytosis (16, 17, 31). Several studies have shown that CD11b is involved in LPS-activated TLR4 signaling pathway (27, 30, 32). However, it was still unknown whether LPS could also induce CD11b endocytosis. To address this issue, BMDMs were treated with LPS/IFN-γ for different time points, and then, the expression of TLR4 and CD11b on the surface of BMDMs was measured. Consistent with previous studies, the expression of TLR4 on the surface of BMDMs was significantly reduced because of TLR4 endocytosis (Figure 6A). Intriguingly, compared with vehicle-treated BMDMs, the expression of CD11b on LPS/IFN-γ-treated BMDMs was also significantly decreased (Figure 6B). To further validate our findings, analogous *in vivo* experiments were performed. Mice stimulated with LPS exhibited significantly reduced levels of TLR4 and CD11b on surface of macrophages obtained from peritoneal cavity (Figures 6C,D) and PBMCs (Figures 6E,F), compared with controls. In order to observe this phenomenon more intuitively, we performed an immunofluorescence experiment. Consistent with above results, fluorescence staining showed that CD11b, in part, underwent endocytosis after LPS/IFN-γ administration (Figure 6G). All these results demonstrated that LPS induced endocytosis of CD11b and TLR4 in macrophages, indicating that CD11b may be involved in the activation of TLR4 signaling pathway.

### Activation of CD11b by LA1 Could Endocytose TLR4 in Macrophages

**Figure 7:**
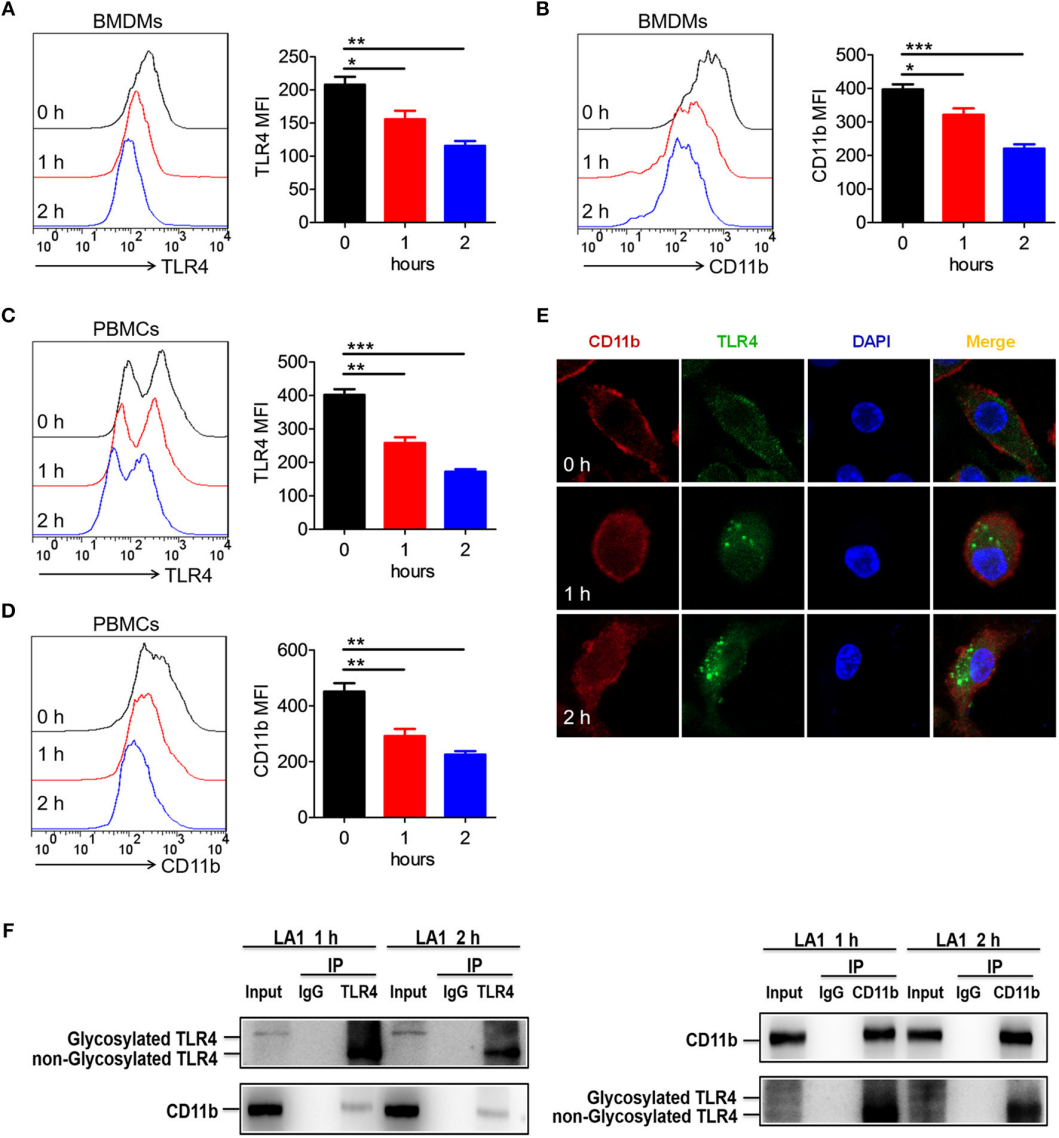
Activation of CD11b by LA1 could endocytose TLR4 in macrophages. **(A, B)** BMDMs were treated with LA1 (20 μM) for 1 and 2 h. FACS analysis was performed to assess the expressions of TLR4 **(A)** and CD11b **(B)** in BMDMs. **(C, D)** C57BL/6 mice were stimulated with LA1 (40 μg/g of body weight) for 1 and 2 h. FACS analysis was performed to assess the expressions of TLR4 **(C)** and CD11b **(D)** in PBMCs. **(E)** Slides containing BMDMs were treated with LA1 (20 μM) for 1 and 2 h, and then, the cells were fixed and permeabilized. The cells were initially stained with anti-TLR4 mAb and anti-CD11b mAb, followed by incubation with an Alexa Fluor 488- and 647-conjugated secondary antibodies. Finally, they were analyzed using a confocal microscope. Red represents CD11b; green represents TLR4; light blue represents nuclei with DAPI; and yellow represents merged TLR4 and CD11b. **(F)** After treated with LA1 (20 μM) for 1 or 2 h, BMDMs were lysed in a modified RIPA buffer. After entrifuged, one aliquot of the supernatant was saved as the input control, the remainder was separately incubated with anti-CD11b antibody, anti-TLR4 antibody and negative control IgG antibody, followed by pull-down with 30 μl Protein A Agarose beads. The beads were then collected by centrifugation and washed three times with cold PBS. The immunoprecipitates were eluted by boiling in loading buffer and subjected to western blot analysis along with input sample as described above. Data shown are representative of three independent experiments. Error bars represent S.E.M. *p < 0.05, **p < 0.01, ***p < 0.001, as determined by one-way analysis of variance (ANOVA) *post-hoc* Tukey Multiple Comparison Test.

**Figure 8:**
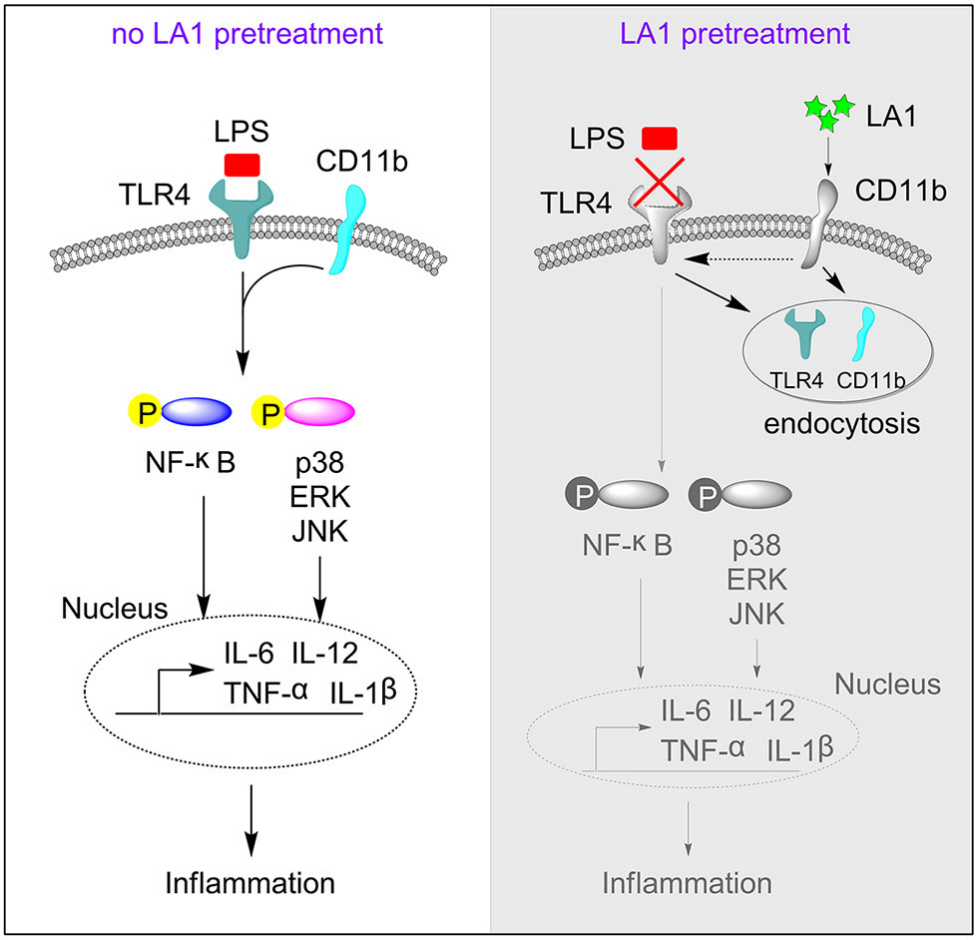
The regulatory mechanism of LA1 on the activation of TLR4 pathway. This schematic shows that activation of CD11b by LA1 induces endocytosis of TLR4 and subsequently, to a certain degree, blocks the interaction of LPS with TLR4 which can decrease activation of TLR4 pathway and inflammation.

Since LA1 inhibited LPS-mediated activation of TLR4 signaling *in vivo* and *in vitro*, we explored the underlying specific mechanisms. We previously proved that LPS induced endocytosis of TLR4 and CD11b, and thus, we investigated whether activation of CD11b by LA1 could endocytose TLR4 and, in turn, inhibit the interaction of LPS and TLR4. In order to further explore our conjecture, the effects of LA1 on the endocytosis of CD11b and TLR4 were investigated *in vitro*. BMDMs were treated with either LA1 or vehicle for different times, and then the expressions of CD11b and TLR4 on surface were detected. Intriguingly, compared with vehicle, LA1 significantly reduced the expressions of CD11b and TLR4 on the surface of BMDMs (Figures 7A,B). Subsequently, we further investigated whether a similar phenomenon occurred *in vivo*. Mice were treated with LA1 for different time intervals. The decrease in CD11b on the surface of peripheral blood cells as well as the decrease of TLR4, suggested that TLR4 is likely to internalize into the cell after being activated by LA1 (Figures 7C,D). Moreover, fluorescence staining and confocal microscopy also confirmed that LA1 administration induced the endocytosis of TLR4 and CD11b (Figure 7E). In addition, CoIP assay was performed to evaluate whether CD11b could interact with TLR4 after activated by LA1. As shown in Figure 7F, the CD11b protein or TLR4 protein was detected in BMDMs after immunoprecipitation with antibodies not only to TLR4 but also to CD11b in the presence of beads. This revealed that CD11b activated by LA1 could interact with TLR4 in macrophages. Taken together, these results confirmed our hypothesis that LA1-mediated activation of CD11b induced the endocytosis of TLR4, and subsequently, inhibited the interaction of LPS with TLR4 partially (Figure 8).

## Discussion

Endotoxic shock is a complex disorder that develops because of dysregulated host response to an infection and it has been widely regarded as a major global health problem (33, 34). In the case of endotoxic shock, inflammatory mediators are produced in excess, resulting in hemodynamic instability and multiple organ failure (35). Therefore, relieving and treating the incidence of endotoxic shock is an important task. Numerous studies have shown that macrophages play an important role in development of endotoxic shock by secreting excessive pro-inflammatory cytokines (36–39). Excessive TLR-activated M1 macrophages contribute to the pathogenesis of inflammatory diseases. Therefore, understanding the intrinsic modulatory mechanisms of M1 macrophage activation will help us understand regulation of innate immune responses and development of inflammatory diseases (40).

Till date, the role of CD11b in regulation of TLR-mediated immune responses remains controversial. On one hand, we used lentivirus to silence the expression of CD11b and found that silencing of CD11b reduced LPS-induced inflammation *in vivo*, which is consistent with another study that reported that inhibition of CD11b-coding gene *itgam* led to low susceptibility to LPS-induced endotoxic shock in mice (22). On the other hand, we found that LA1, which activates CD11b, significantly inhibited LPS-induced pro-inflammatory response in macrophages. It seemed contradictory that silencing as well as activation of CD11b relieved LPS-induced endotoxic shock *in vivo*. As we know, the internal environment was complicated and the silencing experiment was not specific to macrophages alone *in vivo*. Currently, we are focusing on generation of CD11b deficient mice and CD11b transgenic mice to further explore function of CD11b in the pathogenesis of inflammatory diseases. Thus, in this study, we mainly focused on the effect of LA1-activated CD11b on LPS-induced pro-inflammatory response and endotoxic shock.

In the present study, we utilized LA1, a newly developed small molecule allosteric agonist of CD11b/CD18, to activate integrin. The function of LA1 may be complex and multifaceted; therefore, we confirmed our results from different angles and aspects in order to eliminate other possibilities. We found that LA1 decreased the mortalities of mice induced by LPS. Otherwise, it protected mice from endotoxic shock through alleviated pathological injuries of liver and lung. Furthermore, data showed that activation of CD11b by LA1 limited the activation of M1 macrophages in endotoxic shock *in vivo* and inhibited LPS/IFN-γ-induced the activation M1 macrophage *in vitro*. It is important to note that LA1 may play its role by affecting the development and/or function of other immune cells during inflammatory diseases. Therefore, more studies are needed to further investigate the function of LA1 and the specific mechanisms of other immune cells during inflammatory diseases.

Furthermore, we found that mice administered with LA1-treated BMDMs showed fewer pathological lesions than mice administered with vehicle-treated BMDMs. However, we found that there were no significant differences in the expressions of CD86 and CD40 in splenic macrophages obtained from the two groups of mice after LPS stimulation (data not shown). We conjectured that differences in pathological lesions between mice administered with LA1-treated BMDMs and mice administered with vehicle-treated BMDMs were due to exogenously injected BMDMs rather than the macrophages present in the mice themselves.

LPS stimulates TLR4 in macrophages and induces the accumulation of TLR4 in endosomes (13, 15–17, 41). In our research, we found that LPS induced internalization of TLR4 and CD11b. Interestingly, activation of CD11b by LA1 could promote the interaction of CD11b and TLR4, and induce accumulation of TLR4 in endosomes. However, due to limited information regarding this phenomenon, further studies are required to elucidate its specific mechanism.

## Author Contributions

XY, YZ, FY, HZ, XF, QZ, ZL, CL, BW, XL, YY, and FH performed the experiments. QM, JZ, HS, ZN, JM, JD, and XM analyzed the data and generated figures. XY, GD, HX, and CS wrote the manuscript.

### Conflict of Interest Statement

The authors declare that the research was conducted in the absence of any commercial or financial relationships that could be construed as a potential conflict of interest.
