# Supplementary material for: Leukadherin-1-Mediated Activation of CD11b Inhibits LPS-Induced Pro-inflammatory Response in Macrophages and Protects Mice Against Endotoxic Shock by Blocking LPS-TLR4 Interaction
Source: Front Immunol. 2019 Feb 12;10:215. doi: 10.3389/fimmu.2019.00215 (PMC6379471; doi:10.3389/fimmu.2019.00215)
Supplement: Supplementary file 1 [file Data_Sheet_1.pdf]

Supplemental Data

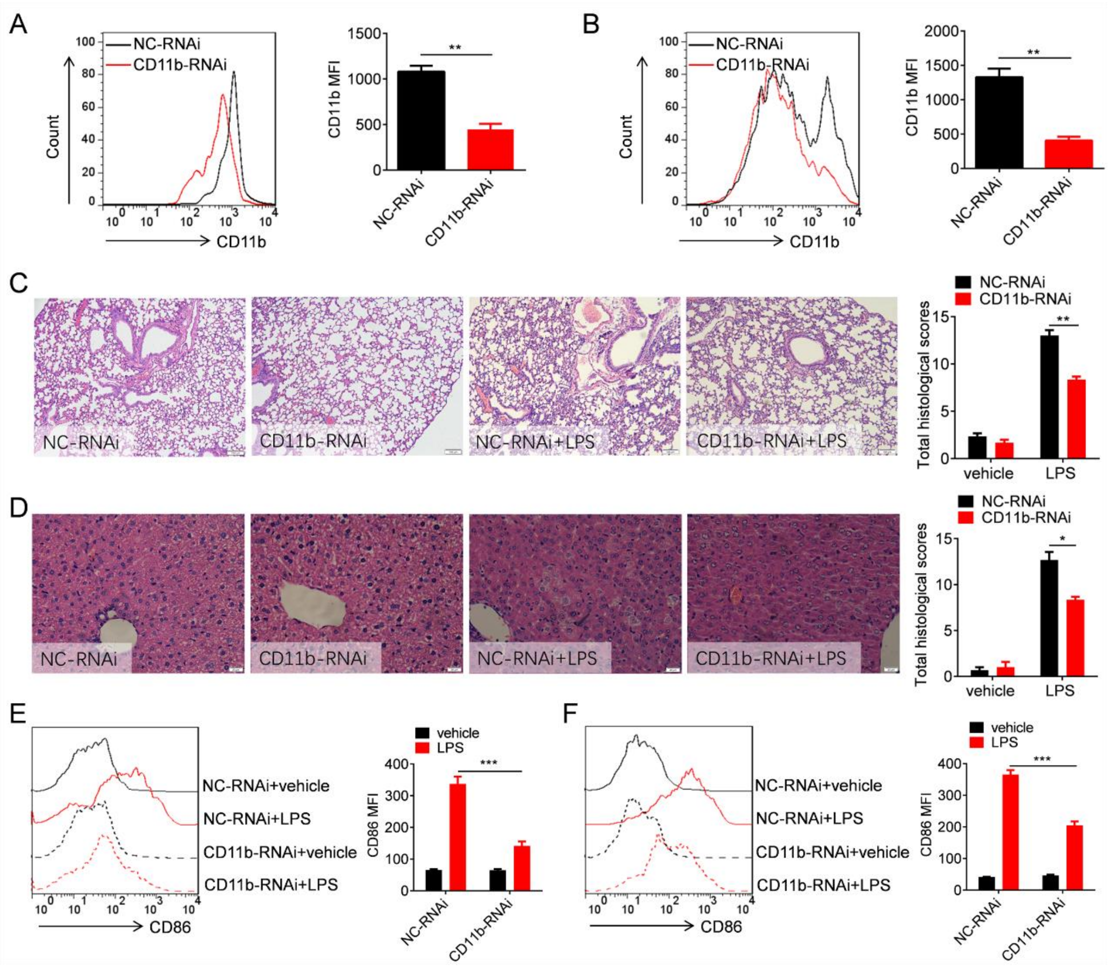

FIGURE S1 | Silencing of CD11b inhibited LPS-induced endotoxin shock and pro-inflammatory response in macrophages *in vivo*. Lentivirus expressing CD11b-specific RNAi (LV-CD11b-RNAi) and lentivirus expressing negative control-specific RNAi (LV-NC-RNAi) were purchased from Genechem (China). For *in vivo* study, C57BL/6 mice were infected with lentivirus expressing CD11b-specific RNAi or negative control-RNAi (1×10^7 TU) by caudal vein. After 1 week, mice were administration with LPS (10 μg/g of body weight). (**A, B**) After 7 days, FACS analysis was performed to assess the CD11b expression in macrophages obtained from peritoneal cavity and spleen. (**C-F**) After 7 days, mice were administration with LPS (10 μg/g of body weight). (**C, D**) Tissues of lung and liver were fixed with 4% paraformaldehyde and paraffin-embedded lung **(C)** and liver **(D)** sections were stained with H&E. (**E, F**) FACS analysis was performed to assess the

expression of CD86 in macrophages and DCs obtained from spleen. The data are shown as the means ± SEM (n=6 replicates) and are representative of three independent experiments. Error bars represent S.E.M. *p < 0.05, **p < 0.01, ***p < 0.001, as determined by Student's t test or two-way ANOVA post-hoc Bonferroni Multiple Comparison Test.

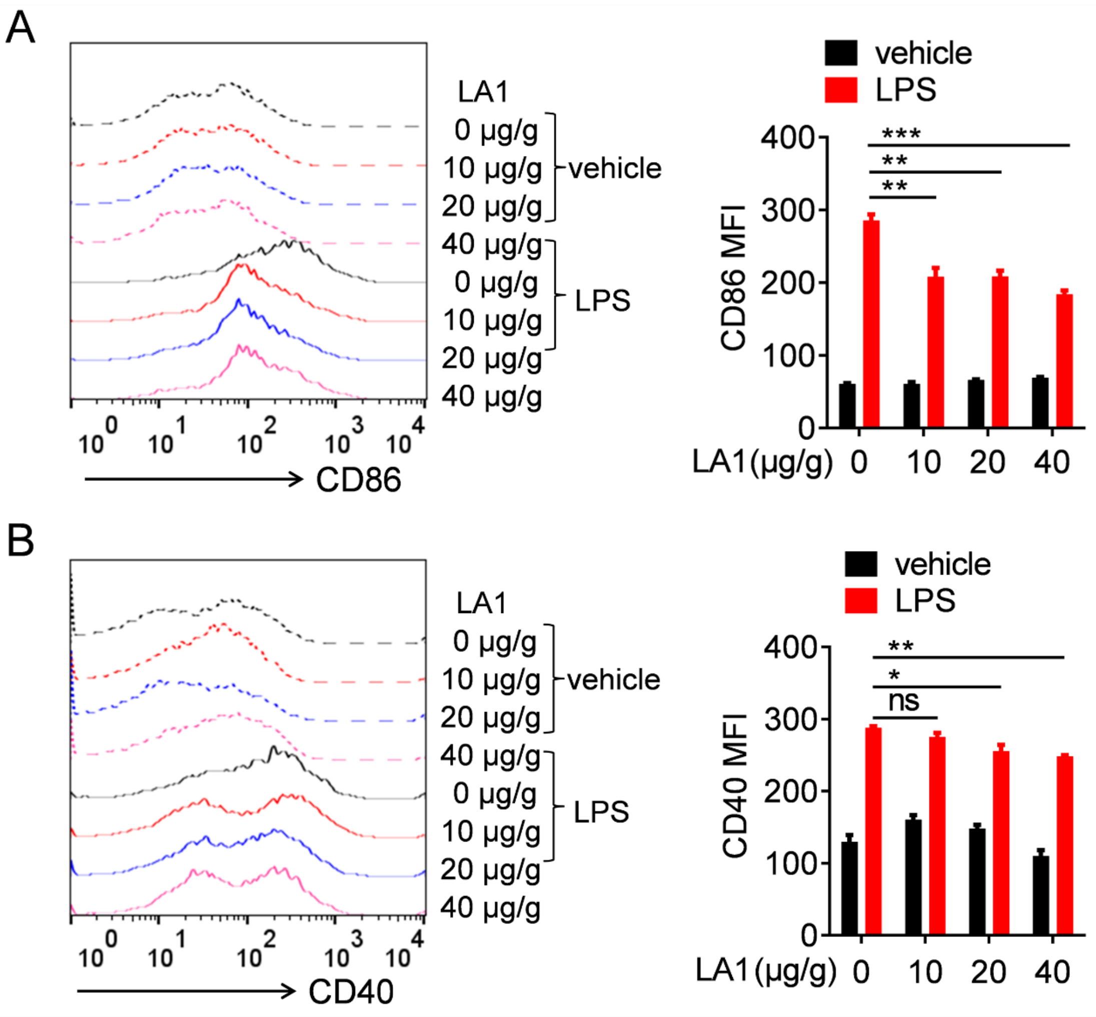

FIGURE S2 | LA1 inhibits the expression of CD86 and CD40 on splenic DCs in endotoxin shock. C57BL/6 mice were treated with LA1 (10, 20, and 40 µg/g of body weight) or vehicle for 2 hours followed by injection of LPS (10 µg/g of body weight). FACS analysis of the CD86 (A) and CD40 (B) expression on splenic DCs at 12 h. The data are shown as the means ± SEM (n=6 replicates) and are representative of three independent experiments. * p < 0.05, ** p < 0.01, *** p < 0.001, as determined by

two-way ANOVA post-hoc Bonferroni Multiple Comparison Test; ns denotes $p > 0.05$.

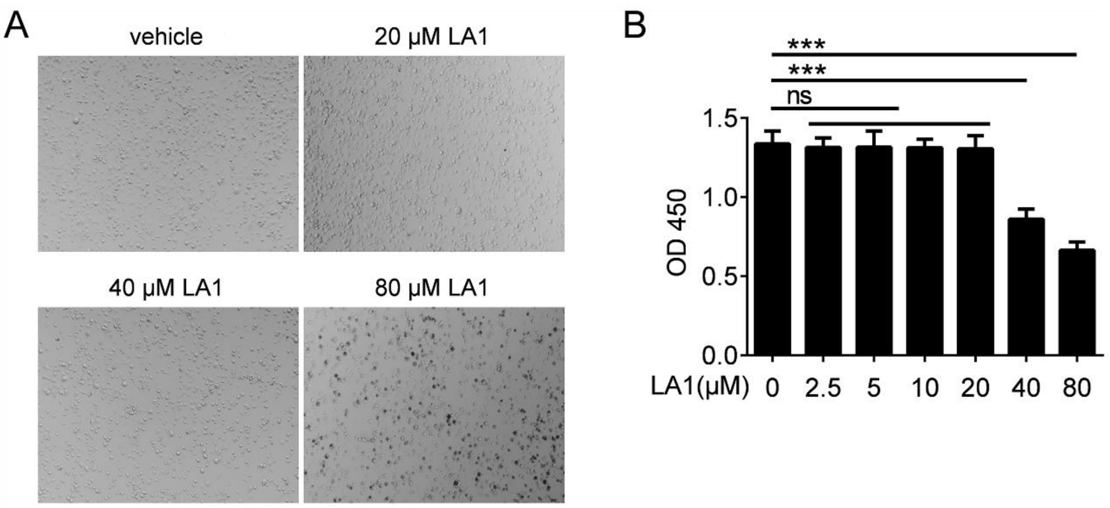

FIGURE S3| The effect of LA1 on the viability of BMDMs. Induction of bone marrow derived macrophages (BMDMs) for 6 days and then the cells were cultured in 96-well cell culture plate (2×10^5/well). (A) One day later, the cells were treated with LA1 (20, 40**,** and 80 μM) for 24 h**.** Cellular morphology was observed by microscope. (B) One day later, BMDMs were treated with LA1 (0, 2.5, 5, 10, 20, 40**,** and 80 μM) for 24 h. Cell counting Kit CCK-8 was applied to measure the viability of BMDMs. Data are representative from one out of three biological replicates, each with three technical replicates. Error bars represent S.E.M. *** $p < 0.001$, as determined by one-way analysis of variance (ANOVA) post-hoc Tukey Multiple Comparison Test.

; ns denotes $p > 0.05$.

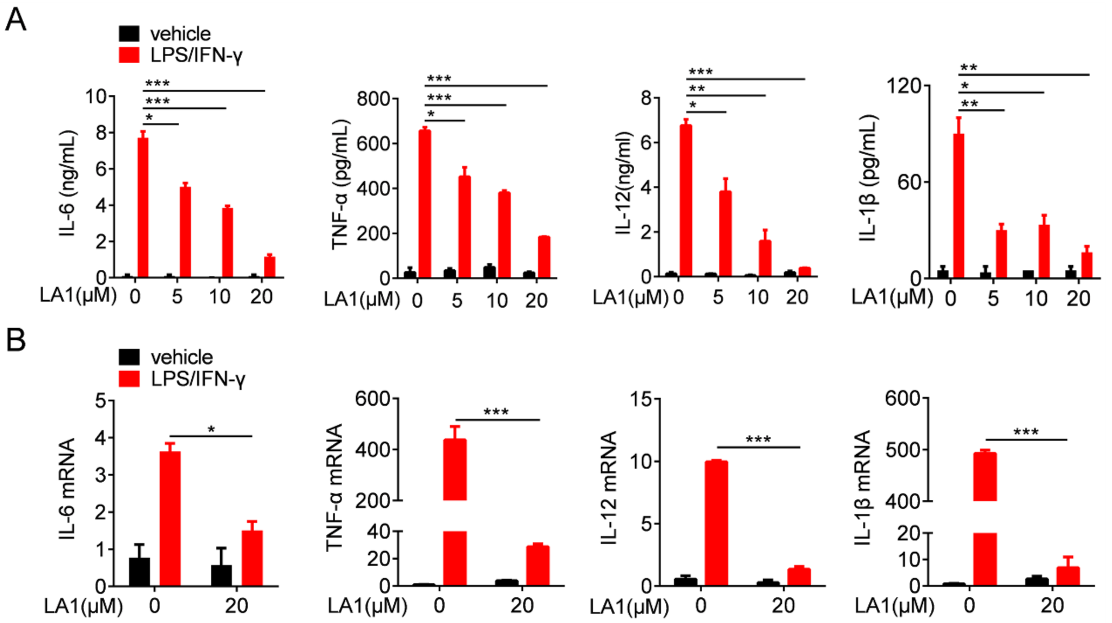

FIGURE S4 | LA1 inhibits LPS plus IFN-γ-induced activation of BMDCs. BMDCs were treated with either LA1 (5, 10, and 20 μM) or vehicle for 2 h followed by stimulation of LPS (200 ng/ml) and IFN-γ (10 ng/ml). **(A)** Levels of IL-6, TNF-α, IL-12 and IL-1β in supernatant were determined by ELISA after 24 h. **(B)** qRT-PCR analyse the expressions of IL-6, TNF-α, IL-12 and IL-1β after 6 h. Data are representative from one out of three biological replicates, each with three technical replicates. Error bars represent S.E.M. * $p < 0.05$, ** $p < 0.01$, *** $p < 0.001$, as determined by two-way ANOVA post-hoc Bonferroni Multiple Comparison Test.
